# Enzymatic Properties of Chitosanase from *Bacillus velezensis* YB1534 and Antibacterial Activity of Its Oligosaccharide Products

**DOI:** 10.3390/foods15030575

**Published:** 2026-02-05

**Authors:** Yiwei Dai, Huiru Zhao, Jincui Wei, Yingxi Chen, Xinping Lin, Sufang Zhang, Chaofan Ji

**Affiliations:** State Key Laboratory of Marine Food Processing & Safety Control, National Engineering Research Center of Seafood, School of Food Science and Technology, Dalian Polytechnic University, Dalian 116034, China; zhaohuiru23@163.com (H.Z.); wjc926825@gmail.com (J.W.); yingxichen24@163.com (Y.C.); yingchaer@163.com (X.L.); zhangsf@dlpu.edu.cn (S.Z.); jichaofan@outlook.com (C.J.)

**Keywords:** chitosanase, heterologous expression, enzyme characterization, pathogen growth inhibition

## Abstract

Chitosan oligosaccharides (COSs), obtained through the hydrolysis of chitosan, exhibit remarkable antibacterial properties. In pursuit of COSs with enhanced antibacterial activity, the enzymatic characteristics of the chitosanase from *Bacillus velezensis* YB1534 (BvChi) were investigated. The purified BvChi displayed optimal activity at pH 6.0 and 50 °C and showed the highest hydrolytic activity using colloidal chitosan as a substrate, with the presence of Mn^2+^. The COSs produced by enzymatic hydrolysis of BvChi exhibited a minimum degree of polymerization (DP) of 2, and their antimicrobial activities against certain pathogenic bacteria (*Escherichia coli*, *Staphylococcus aureus*, *Salmonella typhi* 50071, and *Aeromonas hydrophila*) were evaluated. Among them, the 20 min hydrolysate showed the strongest growth inhibition against all these pathogens, demonstrated by the inhibition zone diameters, and its MIC and MBC values toward *A. hydrophila* were 0.625 and 1.25 mg/mL, respectively. Thin-layer chromatography (TLC) analysis showed that the hydrolyzed products after 20 min contains more COSs with DP > 5. These findings highlighted the potential of BvChi as a biocatalyst for producing antimicrobial COSs, applicable in food preservation and biomedical fields.

## 1. Introduction

Chitin, recognized as the second most prevalent polysaccharide following cellulose, is referred to as “animal cellulose” and constitutes a renewable resource. It is mainly present in the exoskeletons of arthropods like shrimp, crabs, and insects, as well as in some plants, bacteria, and fungi [1,2,3]. Chitosan, derived from the deacetylation of chitin, is a β-1,4-linked linear insoluble polymer composed of D-glucosamine (GlcN) units [4]. While chitosan is a naturally occurring alkaline polysaccharide that is non-toxic and possesses excellent moisturizing and adsorptive properties, its insolubility limits its biological applications [5,6]. Chitosan oligosaccharides (COSs), the hydrolyzed products of chitosan with a low degree of polymerization, demonstrate superior water solubility and biocompatibility compared to chitosan. In addition, COSs also exhibit a broad spectrum of bioactivities, including antimicrobial, anti-inflammatory, antioxidant, anti-photoaging, antitumor, and immune-enhancing effects [6]. Thus, while chitosan finds specific applications in areas such as wound dressing matrices or packaging films where its solid-state properties are advantageous, the enhanced aqueous solubility of COSs makes them uniquely suited for applications requiring bioavailability in solution, such as active ingredients in pharmaceuticals, nutraceuticals, cosmeceuticals, and agrochemicals.

The inherent properties of COSs, including their cationic charge, low molecular weight, and amphiphilic structure, collectively underpin their potent antibacterial activity. Two main mechanisms have been suggested as the cause of the inhibition of microbial cells by COSs [7]. One mechanism involves the protonated amino groups in COSs, which facilitate electrostatic interactions with negatively charged elements of microbial cell membranes, such as teichoic acids in Gram-positive bacteria and lipopolysaccharides in Gram-negative bacteria. This interaction destabilizes the membrane, enhances its permeability, causes leakage of intracellular contents, and ultimately results in bacterial death [8]. Another suggested mechanism for antibacterial activity of COSs is that the cationic COSs can adsorb to negatively charged bacterial DNA, forming COSs-DNA complexes that physically obstruct RNA polymerase binding and transcriptional elongation. This blockade of RNA synthesis effectively silences essential gene expression, leading to impaired bacterial growth and replication [9]. It has been reported that the antibacterial efficacy of COSs is significantly influenced by their degree of polymerization or molecular weight [10]. And COSs with lower molecular weights exhibit enhanced antibacterial efficacy as they can penetrate cells more easily, thereby supporting the proposed transcriptional blockade mechanism [11].

COSs can be produced from chitosan using chitosanases (EC 3.2.1.132), a class of glycosylases that specifically cleave the β-1,4 glycosidic bonds in chitosan with varying degrees of acetylation. These enzymes are naturally present in microorganisms and plant tissues. Based on sequence homology, chitosanases are classified into seven glycoside hydrolase (GH) families: GH3, GH5, GH7, GH8, GH46, GH75, and GH80 [12]. Among these, the GH46 family stands out for its broad membership, extensive research focus, and detailed structural and functional characterization [13]. Chitosanases from the GH46 family, isolated from bacteria such as *Bacillus paramycoides* [14], *Streptomyces hygroscopicus* [15], and *Aquabacterium* sp. [16], are recognized for their higher specificity towards chitosan compared to those from other families.

In a previous study, we isolated and identified the chitosanase-producing strain *Bacillus velezensis* YB1534 from shrimp samples. To elucidate the enzymatic properties of its chitosanase (BvChi) and evaluate the antibacterial potential of its enzymatic hydrolysis products, we heterologously expressed the chitosanase gene from *B. velezensis* YB1534 in *Escherichia coli* BL21 (DE3). Although several chitosanases from *Bacillus* spp. have been heterologously expressed in *E. coli* [17,18], the expression of chitosanase from *B. velezensis* YB1534 in *E. coli* BL21(DE3) remains unexplored. The antibacterial activities of the hydrolyzed products toward three common pathogenic bacteria (*E. coli*, *S. aureus*, and *S. typhi* 50071), and one typical pathogenic bacterium in aquatic products (*A. hydrophila*), were investigated. This study provides novel insights into green antibacterial strategies for aquatic products by developing a biocatalytic approach for sustainable production of COSs with targeted antimicrobial activity.

## 2. Materials and Methods

### 2.1. Chemical Reagents

LB medium, isopropyl β-D-1-thiogalactopyranoside (IPTG), and ampicillin (AMP) were obtained from Sangon Biotech Co., Ltd. (Shanghai, China). COS standards (DP 3-7) were purchased from Yuanye Bio-Tech Co., Ltd. (Shanghai, China). GlcN, glucan (derived from *Leukonostoc* sp.; M_W_: 50,000 Da), and chitosan (derived from shrimp and crab shells; M_W_: 126,000–265,000 Da; degree of deacetylation (DDA) ≥ 95%) were obtained from Macklin reagent Co., Ltd. (Shanghai, China). Chitin (derived from shrimp shells) and sodium carboxymethyl cellulose were supplied by Aladdin Co., Ltd. (Shanghai, China). 3,5-dinitrosalicylic acid (DNS) reagent was obtained from Solarbio Science & Technology Co., Ltd. (Beijing, China).

### 2.2. Strains and Plasmids

*B. velezensis* YB1534 was previously isolated from shrimp in our laboratory and preserved in the Guangdong Provincial Microbial Strain Preservation Center (GDMCC 65995). *E. coli*, *S. aureus*, *S. typhi* 50071, and *A. hydrophila* were used to test the antibacterial activity of enzymatic hydrolysate. The host strain *E. coli* BL21 (DE3) (Sangon, Shanghai, China) was selected for gene cloning and protein expression. The plasmid pET22b was employed to achieve heterologous expression of chitosanase in this strain.

### 2.3. Cloning, Expression, and Purification of Chitosanase BvChi

The gene sequence and amino acid sequence of *B. velezensis* YB1534 were published in Genebank (NCBI accession number: NZ_AJST01000001.1 and WP_029325847.1). Genomic DNA was isolated from *B. velezensis* YB1534 using a Genomic DNA Isolation Kit (Sangon, Shanghai, China). The gene encoding BvChi was PCR-amplified from the genomic DNA of *B. velezensis* YB1534 and ligated into the plasmid pET22b(+) using the ClonExpress^®^ II One Step Cloning Kit (Vazyme, Nanjing, China), following the manufacturer’s instructions (primers listed in Table 1). The recombinant plasmid named pET22b-*BvChi* was then transformed into *E. coli* BL21 (DE3). The positive transformant was designated *E. coli* BL21/pET22b-*BvChi* and stored at −80 °C for further studies.

The engineered *E. coli* strains with the BvChi gene were cultured in LB medium with 50 μg/mL AMP at 37 °C. When OD_600_ reached 0.6, 1 mM IPTG was added to induce the expression of heterologous protein. The induction was conducted at 20, 25, and 30 °C, respectively, and the extracellular enzymatic activities at different induction times (0–24 h) were investigated. After induction, the cell-free supernatant was collected and purified using Ni^2+^-nitrilotriacetic acid (NTA) resin (Yuanye Bio-Tech, Shanghai, China) according to manufacturer’s instructions. Purified enzymes were quantified using a NanoDrop 2000c spectrophotometer (Thermo Fisher Scientific, Waltham, MA, USA), analyzed by SDS-PAGE on 12% acrylamide gel, and stored at 4 °C for further use.

### 2.4. Sequence Analysis

A phylogenetic tree was constructed based on amino acid sequences of chitosanase using the neighbor-joining method implemented in MEGA software (Version 6.0). Sequence alignments of the amino acids of BvChi and other reported chitosanase belonging to the GH46 family were performed with GeneDoc software (Version 2.7.0) programs.

### 2.5. Chitosanase Activity Assay

BvChi activity was quantified by measuring the release of reducing sugars derived from the hydrolysis of chitosan or related polysaccharides using the DNS method. The reaction was conducted in a 2 mL system at 50 °C, containing 50 mM HAc-NaAc buffer (pH 6.0), 1% (*w*/*v*) colloidal chitosan substrate (prepared in 0.2 M sodium acetate buffer, pH 5.5, and preincubated at 50 °C for 5 min), and 500 μL purified BvChi enzyme. After 20 min of incubation, the reaction was terminated by boiling for 10 min. Subsequently, 3 mL of DNS reagent was added to each sample, followed by heating at 100 °C for 10 min and rapid cooling on ice for 2 min. The mixtures were then centrifuged at 12,000× *g* for 5 min to precipitate unreacted chitosan. The absorbance of the resulting supernatants was measured at 540 nm. One unit of enzyme activity was defined as the amount of enzyme that releases 1 μmol reducing ends (as GlcN equivalents) per minute, as determined by the DNS assay.

### 2.6. Enzymatic Properties of BvChi

To evaluate the effect of temperature on BvChi activity, reactions were performed with 1% (*w*/*v*) colloidal chitosan substrate in 50 mM HAc-NaAc buffer (pH 6.0) at various temperatures (30, 40, 50, 60, 70, and 80 °C), maintaining all other parameters consistent with the standard assay. For thermal stability analysis, purified BvChi (3.38 nM final concentration in 2 mL total volume) was pre-incubated in the same buffer at 40, 50, or 60 °C for 0–4.5 h. Residual enzymatic activity was subsequently measured under standard conditions using the DNS method.

The pH effect on BvChi activity was evaluated using 1% (*w*/*v*) colloidal chitosan at 50 °C across a broad pH range (4.0–8.0) with three buffer systems: 50 mM HAc-NaAc (pH 3.0–6.0), 50 mM PBS (pH 6.0–8.0), and 50 mM Tris-HCl (pH 8.0–10.0). For pH stability analysis, the enzyme was stored in different pH buffers at 4 °C for 2 h, and residual activities were measured via the DNS method.

To investigate the substrate specificity of BvChi, enzymatic activity assays were conducted at 50 °C in 50 mM HAc-NaAc buffer (pH 6.0) using five polysaccharides at 1% (*w*/*v*) concentration: colloidal chitosan, water-soluble chitosan, colloidal chitin, sodium carboxymethyl cellulose, and glucan. The relative activity was determined under the same conditions as listed above.

To determine the effect of metal ions on the hydrolysis activity of BvChi, the reaction was performed in the presence of 1 mM, 5 mM, and 10 mM of several common metal ions, including Mn^2+^, Zn^2+^, Mg^2+^, Ba^2+^, Ca^2+^, Fe^3+^, and Cu^2+^. Other conditions were the same as in the enzyme assay.

Kinetic parameters were determined using colloidal chitosan concentrations ranging from 0.2% to 1.6% (*w*/*v*) under standard assay conditions. The kinetic parameter (*K_m_*) and maximum reaction rate (*V_max_*) of BvChi were calculated by Hanes–Woolf plot.

All experiments were performed in triplicate (*n* = 3) on independent biological samples. Data are presented as mean ± standard deviation (SD). The same experimental replication and data presentation were applied to all assays described in this section. Significance analysis was performed using Student’s *t*-test (*t*-test) with * indicating *p*-value ≤ 0.05, ** indicating *p*-value ≤ 0.01 and *** indicating *p*-value ≤ 0.001.

### 2.7. Hydrolyzed Products Analysis by TLC

The enzymatic products of BvChi were analyzed by thin-layer chromatography (TLC) using silica gel 60 F254 glass plates (Merck, Darmstadt, Germany). Hydrolysates collected at 0, 20, 40, and 60 min reaction intervals were spotted onto the plates, which were developed with a solvent system consisting of n-butanol/acetic acid/water/ammonium hydroxide (26:13:13:2.6, *v*/*v*/*v*/*v*). After development, the plates were sprayed with a colorimetric reagent (diphenylamine: phenylamine: acetone: phosphoric acid = 1 g:1 mL:50 mL:5 mL) and heated at 110 °C for 10 min to visualize brown-colored product spots.

### 2.8. Antibacterial Activities of the COSs Products

The anti-bactericidal effect of chitosan hydrolysates was examined against the strains of *E. coli*, *S. aureus*, *S. typhi* 50071, and *A. hydrophila*. Among these, *E. coli*, *S. aureus*, and *S. typhi* 50071 were laboratory-preserved strains, while *A. hydrophila* was isolated from marine sediment. These indicator bacteria were cultured in LB medium at 37 °C for 24 h. Based on the hemocytometer counts, the bacterial suspensions were further diluted with sterile physiological saline to achieve a final concentration of 1.0 × 10^7^ CFU/mL, which was subsequently used as standardized bacterial suspension.

Hydrolysates of BvChi were collected at 0, 20, 40, and 60 min reaction intervals, and the inhibitory effects were evaluated against four pathogenic bacteria using the agar diffusion disk method [19]. Sterilized filter paper disks (6 mm diameter) were saturated with 20 µL of hydrolyzed samples and placed onto agar plates pre-inoculated with test organisms at 37 °C. Antimicrobial activity was quantified by measuring the diameter of clear inhibition zones around the disks after 12 h incubation [20]. The antibacterial test was performed in triplicate.

The hydrolysate from the 20 min reaction was heated at 100 °C for 10 min and then centrifuged at 12,000× *g* for 5 min to remove the enzyme and residual chitosan. The prepared solution was lyophilized and then redissolved in deionized water to achieve a concentration range of 0.039–5 mg/mL. Antibacterial assays were conducted in 96-well polystyrene culture plates using the 2-fold serial dilution method. The tested bacterial cultures were pre-cultivated overnight in LB broth at 37 °C with shaking at 220 rpm. Then, grown bacteria were diluted by sterile LB broth to OD_600_ = 0.5, and 100 μL of prepared bacterial suspensions were filled into 96-well polystyrene cultural plates. Suitable quantities of tested COSs were added to the aliquots and adjusted to final volume (200 μL) with sterile LB broth [21]. After thorough mixing, the tubes were incubated at 37 °C for 24 h under aerobic conditions. All MIC and MBC determinations were performed in triplicate in independent experiments. The MIC was defined as the lowest concentration of the extract exhibiting no visible turbidity or cloudiness compared to the growth control. To confirm the MBC, the tubes showing no turbidity were subcultured onto LB agar plates. The plates were then incubated at 37 °C for an additional 24 h. The MBC was recorded as the lowest concentration of the extract that completely inhibited bacterial growth on the LB agar plates.

## 3. Results and Discussion

### 3.1. Gene Cloning, Overexpression, and Protein Purification

The chitosan-degrading strains were screened from shrimp using the plate transparent circle method. A total of 17 strains were initially screened and identified based on colony morphology and 16S rRNA gene sequencing (Table S1). The chitosan-degrading activities were further investigated using the DNS method. Among these strains, *B. velezensis* YB1534 exhibited the highest hydrolytic activity (7.23 ± 0.27 U/mL) (Figure S1). The whole-genome sequence of *B. velezensis* available in the NCBI database indicated that it contains a gene encoding chitosanase. Therefore, *B. velezensis* YB1534 was selected as the source strain for chitosanase gene cloning and functional characterization.

The BvChi gene was amplified from the genomic DNA of *B. velezensis* YB1534 and cloned into the expression vector pET-22b. Sequence analysis revealed an 837 bp open reading frame encoding a 278-amino-acid polypeptide with a calculated molecular mass of 31.4 kDa and an isoelectric point (pI) of 9.05. The whole-DNA and amino acid sequences of BvChi are presented in Figure S2. SignalP prediction (https://services.healthtech.dtu.dk/services/SignalP-5.0/, accessed on 11 November 2025) identified a 35-residue signal peptide at the N-terminus, indicating that BvChi is secreted extracellularly (Figure S3).

**Figure 1:**
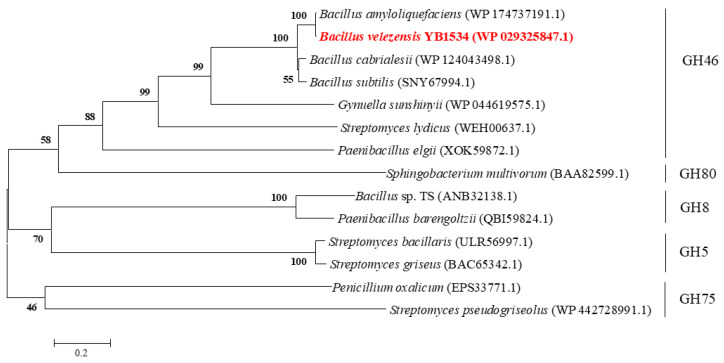
The phylogenetic tree was constructed by the neighbor-joining algorithm in MEGA6 and was based on the amino acid sequence alignment BvChi and chitosanases with GH46, GH80, GH8, GH5, and GH75. BvChi is in red bold font.

**Figure 2:**
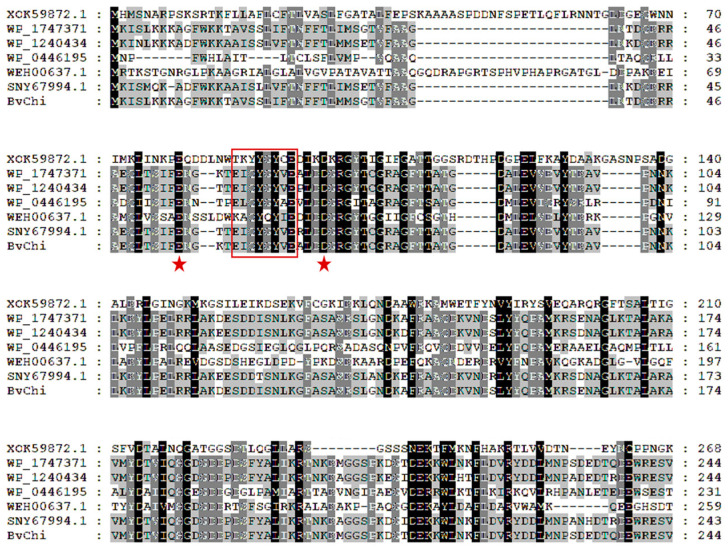
Multisequence alignment analysis of BvChi and other characterized GH46 members. The highly conserved catalytic sites are labeled with a red star (★).

In order to classify the BvChi within the broader context of chitosanases, a phylogenetic tree was constructed. A phylogenetic tree was constructed containing BvChi and other chitosanases from GH46, GH5, GH75, GH8, and GH80 (Figure 1). The phylogenetic analysis indicated that BvChi belongs to the GH46 family. BvChi was aligned with other GH46 family chitosanases, including *Paenibacillus elgii* (XOK59872.1), *Bacillus amyloliquefaciens* (WP174737191.1), *Bacillus cabrialesii* (WP124043498.1), *Gynuella sunshinyii* (WP044619575.1), *Streptomyces lydicus* (WEH00637.1), and *Bacillus subtilis* (SNY67994.1). The multiple sequence alignment is displayed in Figure 2. Based on the results of alignment and previous studies [22,23], two amino acids (E55 and D71) were considered as catalytic active sites. These acidic amino acids may promote the cleavage of glycosidic bonds by providing protons or accepting protons and stabilize the substrate-binding site to allow the substrate to undergo catalysis [23].

**Figure 3:**
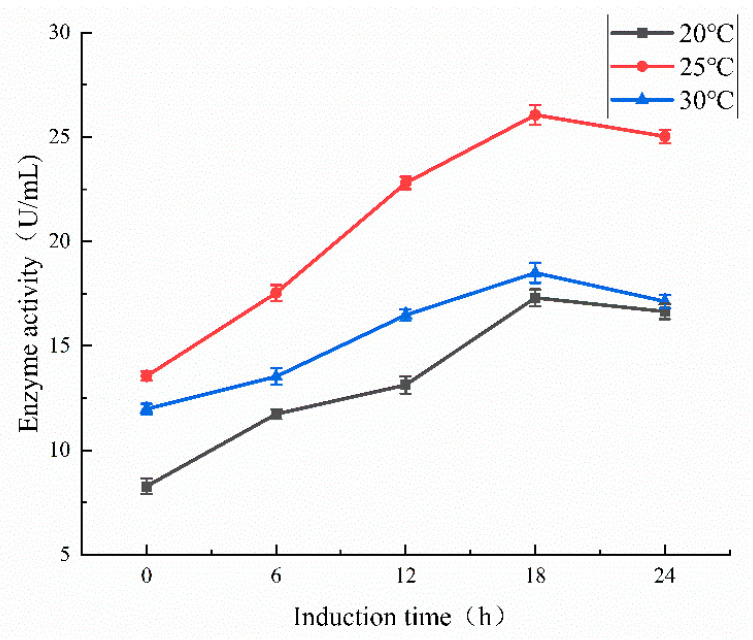
The extracellular activities of BvChi at different induction times.

To determine the optimal induction temperature for enzyme production, the recombinant strain *E. coli* BL21(DE3) harboring pET22b-BvChi was cultured at three temperatures (20 °C, 25 °C, 30 °C) with 1 mM IPTG addition. The extracellular enzyme activity was quantified at 6 h intervals over 24 h using DNS assay (Figure 3). The basal chitosanase activity observed at 0 h induction time strongly indicated that BvChi encodes a chitosanase with constitutive transcriptional activity. However, supplementation with 1 mM IPTG led to a marked increase in extracellular enzyme activity, indicating that the promoter also exhibits partial inducibility under the tested culture conditions. A similar observation was reported by Aktuganov et al. [24], which may be explained by the leaky expression commonly associated with T7-based expression systems in *E. coli* [25].

The highest extracellular activity of BvChi (26.06 ± 0.47 U/mL) was achieved at 18 h induction, representing a 1.4-fold and 1.5-fold increase compared to 30 °C (18.50 ± 0.48 U/mL) and 20 °C (17.30 ± 0.41 U/mL), respectively. This differential response to induction temperature can be attributed to two counteracting physiological effects. On the one hand, higher induction temperatures typically promote faster protein synthesis and cellular metabolism, but in this case, the rapid accumulation of recombinant protein in the cytoplasm may have led to improper folding or aggregation into insoluble inclusion bodies, thereby reducing the yield of active extracellular enzyme. On the other hand, although lower temperatures are generally favorable for proper protein folding and solubility, the marked suppression of cellular growth observed at 20 °C likely limited overall protein synthesis capacity and consequently lowered total enzyme production. Thus, the optimal temperature of 25 °C appears to represent a balance between adequate cellular metabolism and proper folding of the recombinant chitosanase.

**Figure 4:**
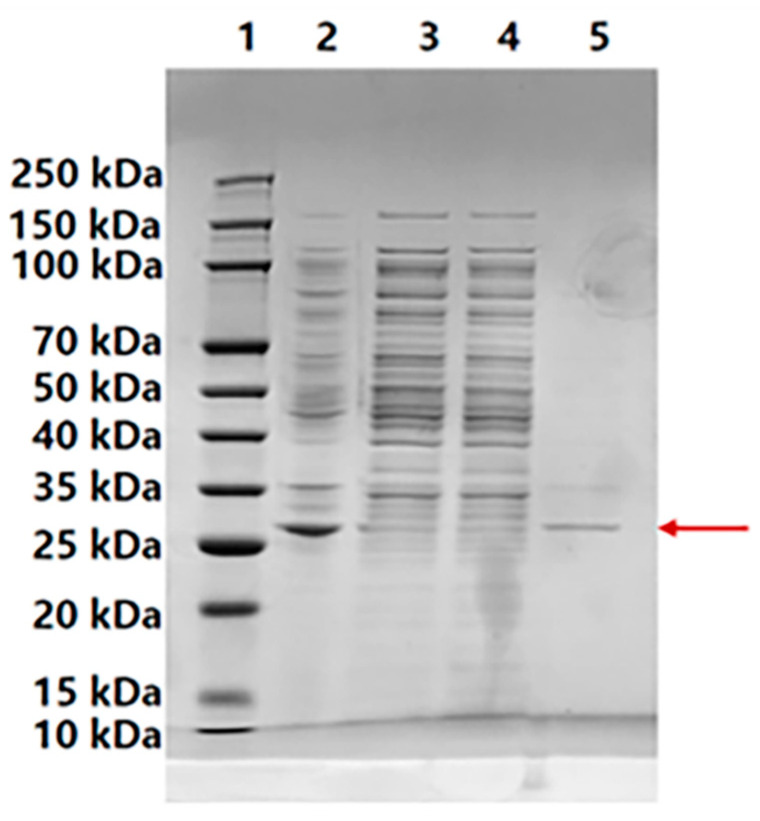
SDS-PAGE results of recombinant BvChi. Line 1, marker; line 2, crude enzyme; line 3, eluent from binding buffer; line 4, eluent from washing buffer; line 5, purified enzyme. The red arrow indicates the position of BvChi on the SDS-PAGE gel.

In our experiment, we observed that the extracellular secretion level of Bvchi was comparatively low, probably attributed to the fact that the host bacterium *E. coli* retained the heterologous expressed enzyme within the periplasmic space. To obtain sufficient protein for detailed enzymatic characterization, the recombinant enzyme was purified from the intracellular fraction. After cell disruption, BvChi was isolated using Ni-affinity chromatography. The purified enzyme was analyzed by SDS-PAGE. As shown in Figure 4, BvChi migrated as a single band with a molecular mass of 31 kDa, which was in accordance with the predicted molecular weight. This result confirmed the successful purification of the recombinant BvChi and supported its use in subsequent biochemical assays.

### 3.2. Enzymatic Characterization of BvChi

**Figure 5:**
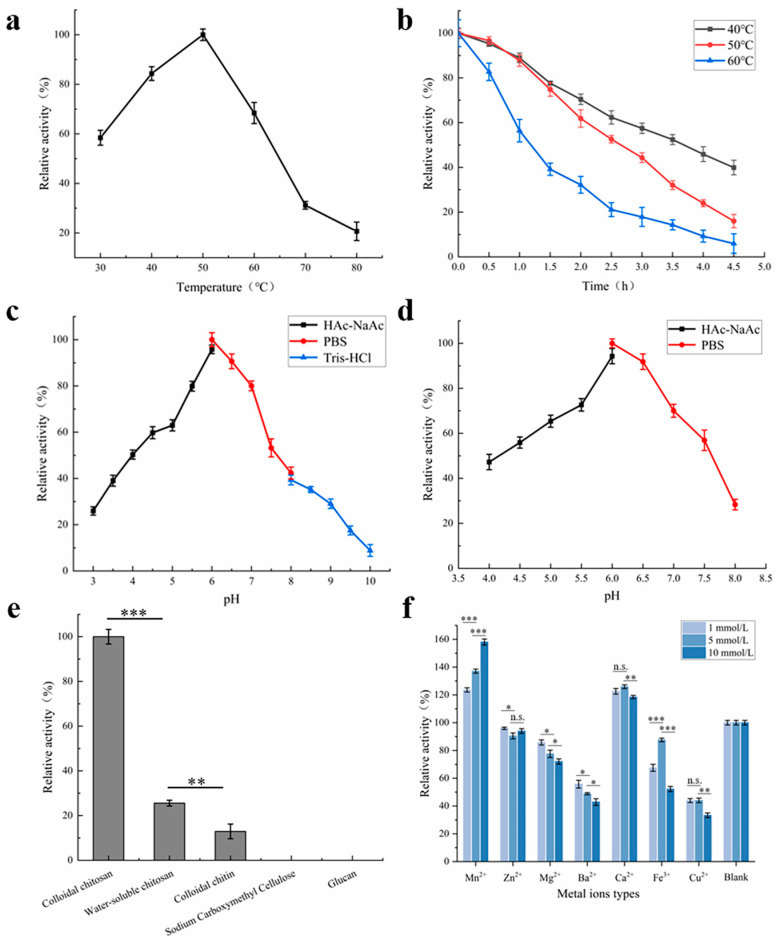
Enzymatic properties of BvChi. (**a**) Effect of temperature on enzyme activity. (**b**) Effect of temperature on enzyme stability. (**c**) Effect of pH on enzyme activity. (**d**) Effect of pH on enzyme stability. (**e**) Effect of substrates on enzyme activity. (**f**) Effect of metal ions on enzyme activity. n.s., not significant; * *p* < 0.05; ** *p* < 0.01; *** *p* < 0.001.

The enzymatic properties of BvChi were systematically investigated through a series of biochemical assays. The optimal temperature for BvChi activity was identified as 50 °C, with a sharp decline in activity observed at temperatures exceeding 60 °C, showing cold adapted characters (Figure 5a). In terms of thermal stability, the enzyme retained over 50% activity after 4 h at 40 °C but exhibited significant inactivation at 60 °C within the same duration (Figure 5b).

The pH activity profile revealed that BvChi exhibited maximal activity at pH 6.0, with broad adaptability across slightly acidic to neutral conditions (Figure 5c,d). Substrate specificity analysis demonstrated a marked preference for colloidal chitosan, while water-soluble chitosan and colloidal chitin showed substantially lower catalytic efficiency. In addition, sodium carboxymethyl cellulose and glucan cannot react with BvChi (Figure 5e).

**Figure 6:**
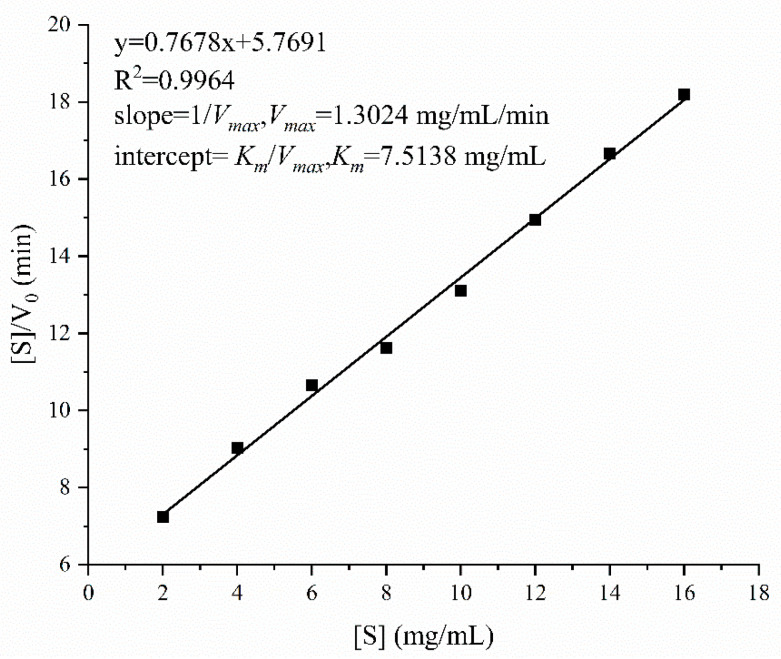
Kinetic parameters of recombinant BvChi.

The enzyme activity of BvChi was also modulated by metal ions. The results displayed that Mn^2+^ and Ca^2+^ significantly enhanced BvChi activity across all tested concentrations, whereas Zn^2+^, Mg^2+^, Ba^2+^, Fe^2+^, and Cu^2+^ exhibited inhibitory effects (Figure 5f). The stimulatory effect of Mn^2+^ on BvChi activity intensified in a concentration-dependent manner. And other chitosanases from different source also showed enhanced activity with the presence of Mn^2+^, demonstrating a potential conserved mechanism of Mn^2+^-mediated activation among chitosanases [14,26]. The hydrolysis of colloidal chitosan by BvChi was assayed at 50 °C and pH 6.0 to evaluate substrate concentration effects. Kinetic parameters determined under these conditions showed that *K_m_* and *V_max_* values of BvChi were 7.5138 mg/mL and 1.3024 mg/mL/min, respectively (Figure 6).

### 3.3. TLC Results of BvChi Hydrolysates

**Figure 7:**
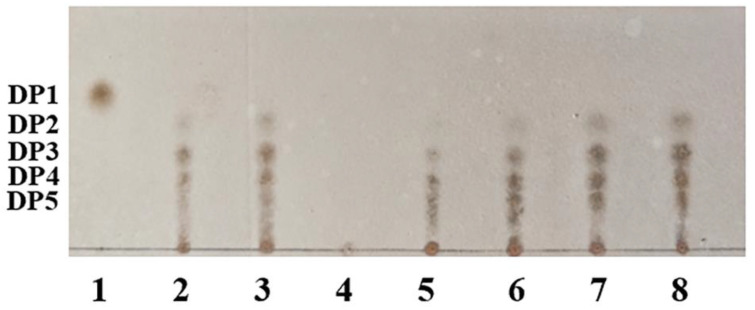
TLC analysis of the hydrolysate of BvChi. 1, 5 mg/mL GlcN standard; 2, 5 mg/mL DP 3-7 COSs standard; 3, 10 mg/mL DP 3-7 COSs standard; 4, enzymatic reaction for 0 min; 5, enzymatic reaction for 20 min; 6, enzymatic reaction for 30 min; 7, enzymatic reaction for 40 min; 8, enzymatic reaction for 60 min.

To investigate the antibacterial mechanisms of hydrolyzed products with different reaction times, the enzymatic hydrolysates of BvChi using colloidal chitosan (1%, *w*/*v*) were analyzed through TLC. TLC analysis revealed a time-dependent shift in the hydrolysate composition (Figure 7). The minimum DP observed was 2, which is identical to that of the hydrolysis products from other enzymes (Table S2) [14,16,24,26,27,28,29]. Hydrolysates generated within 30 min contained a higher proportion of COSs with DP>5, while continued enzymatic hydrolysis led to the progressive accumulation of DP2 and DP3, as indicated by increasing spot intensities. The early hydrolysates (e.g., at 20 min) are likely enriched in COSs with DP6-8, which are reported to possess superior antibacterial activity due to optimal interaction with bacterial cell walls [30], and this aligns with the finding that a minimal DP of 6 is required for significant antimicrobial effects [31]. This provided a plausible explanation for their potent antibacterial activity. However, the semi-quantitative nature of TLC precludes a definitive correlation between the DP distribution and the activity profile. The maximal activity observed for the 20 min hydrolysate may also arise from synergistic effects between multiple COS fractions present in the mixture [32], rather than from a single DP class. To precisely quantify the DP distribution and elucidate the structure–activity relationship, further analysis using quantitative techniques such as HPLC or NMR is warranted.

### 3.4. Antibacterial Activity of Hydrolyzed Products

**Figure 8:**
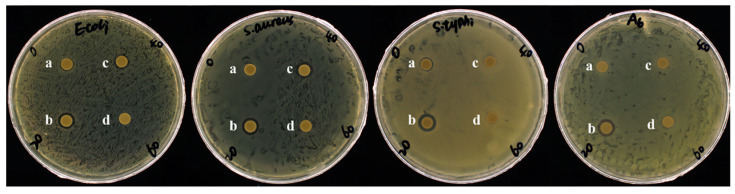
The inhibitory effects of hydrolysis products with different reaction times (a: 0 min; b: 20 min; c: 40 min; d: 60 min) toward four types of pathogenic bacteria (from left to right: *E. coli*, *S. aureus*, *S. typhi* 50071 and *A. hydrophila*).

The antibacterial activities of hydrolysate produced by BvChi against four pathogenic bacteria was evaluated via the disk filter paper assay. The results demonstrated that the hydrolyzed products exhibited varying degrees of inhibitory effects on all pathogenic bacteria (Figure 8). Before enzymatic hydrolysis, the presence of inhibition zones in the *E. coli* and *S. typhi* 50071 groups indicated that the 1% colloid chitosan in the reaction substrate exhibited inhibitory effects against these two pathogens. Notably, after 20 min of hydrolysis, the inhibition zones reached their maximum diameters across all tested pathogens, with the highest values observed for *S. aureus* (3.14 ± 0.06 mm), *S. typhi* 50071 (3.49 ± 0.13 mm), and *A. hydrophila* (3.63 ± 0.05 mm), indicating a peak in antibacterial efficacy during this reaction time (Table 2). However, prolonged hydrolysis time led to a decline in antibacterial activity, with inhibition zones decreasing to baseline levels for *E. coli* and *S. aureus* after reacting for 60 min. These findings suggest that the hydrolyzed products possess time-dependent antibacterial properties, with optimal activity occurring at 20 min of hydrolysis.

Compared to the antimicrobial effects observed in the agar well diffusion assay, the inhibition zones observed in this assay were relatively modest in diameter compared to those reported for certain commercial antibiotics or other well-characterized antimicrobial agents. This may be attributed to several methodological and compositional factors. First, the assay was performed using the crude enzymatic hydrolysate without prior concentration, which likely resulted in a lower effective concentration of bioactive COSs in the diffusion medium. Second, the molecular size and charge distribution of the COS generated under the tested conditions may influence their diffusion rate through the agar matrix. Larger or more aggregated oligomers might diffuse more slowly, leading to smaller visible zones despite possessing intrinsic inhibitory activity [33]. Finally, the primarily bacteriostatic mode of action associated with chitosan-derived oligosaccharides—often involving membrane disruption and leakage rather than immediate cell lysis—might not produce as sharply defined or extensive zones of clearance as rapidly bactericidal compounds [34,35]. Nevertheless, the consistent and reproducible inhibition observed across all pathogens validated the broad-spectrum potential of the BvChi-generated hydrolysate and supported its further investigation as a bioactive agent for applications in which sustained microbial suppression is desired, such as in food preservation or antimicrobial coatings.

The antimicrobial efficacy of the 20 min chitosan hydrolysate generated by BvChi was quantitatively assessed using MIC and MBC assays (Table 3). Results indicated differential susceptibility among the tested bacterial strains. Specifically, the hydrolysate exhibited a MIC of 0.625 mg/mL against *E. coli* and *A. hydrophila*, demonstrating its capacity to inhibit bacterial growth at this concentration. However, a higher concentration of 1.25 mg/mL was necessary to achieve a bactericidal effect, as reflected by the MBC value, suggesting that while growth suppression occurs at lower concentrations, complete eradication of *E. coli* and *A. hydrophila* requires elevated doses. Among these tested strains, *S. aureus* exhibited the lowest MIC and MBC values. As the only Gram-positive bacterium examined, *S. aureus* appears to be the most sensitive to COSs. It has been reported that the positively charged glucosamine units in COSs promote binding to negatively charged carboxyl groups on the Gram-positive bacterial cell wall, leading to suppression of bacterial growth [36]. Notably, *S. typhi* 50071 emerged as the most resistant strain, requiring 1.25 mg/mL for MIC and 2.5 mg/mL for MBC. This increased tolerance may be attributed to its unique outer membrane structure and efflux pump systems, which are common in Gram-negative bacteria, along with potential specific adaptations such as reduced membrane permeability or enhanced enzymatic degradation of chitosan oligomers [37].

These results collectively suggested that the chitosan hydrolysate produced by BvChi possessed broad-spectrum antibacterial activity, albeit with strain-specific potency. The bactericidal effect against *S. aureus* at lower concentrations highlighted its potential for targeting pathogens in food processing where complete microbial elimination is critical. Meanwhile, the effective suppression of *A. hydrophila* supported its applicability in food systems, including aquatic preservation and food safety contexts. Further structural and mechanistic studies on the active components of chitosan hydrolysate could elucidate the basis for this differential activity and inform its optimized use in antimicrobial formulations.

**Table 3:** Results of MIC and MBC (mg/mL) of 20 min hydrolyzed products against the bacterial strains.

Strain	MIC	MBC
*E. coli*	0.625	1.25
*S. aureus*	0.313	0.625
*S. typhi* 50071	1.25	2.5
*A. hydrophila*	0.625	1.25

## 4. Conclusions

A chitosanase gene (BvChi) from *B. velezensis* YB1534 was successfully identified and heterologously expressed in *E. coli* BL21. The purified BvChi enzyme exhibited optimal activity at 50 °C and pH 6.0 when using 1% colloidal chitosan as the substrate. Notably, the addition of Mn^2+^ and Ca^2+^ ions enhanced the enzymatic activity of BvChi. The hydrolytic products generated by BvChi demonstrated significant antibacterial activity against several pathogenic bacteria, including *E. coli*, *S. aureus*, *S. typhi* 50071, and *A. hydrophila*. Among these products, the 20 min hydrolysate displayed the highest inhibitory effect, which may be associated with its relatively higher content of COSs with DP > 5. These findings provide a theoretical foundation for the potential application of chitosanase hydrolysates in the preservation of aquatic products. However, the antibacterial mechanism of COSs with DP6-8 remains incompletely understood, necessitating further investigation to fully explore their potential for commercial use in food preservation.

## Figures and Tables

**Table 1:** The primers designed for amplification of the chitosanase BvChi gene.

Primer	Sequence (5′-3′)
BvChi-F	aagaaggagatatacatatgATGAAAATCAGCTTGAAGAAAAAAGCAG
BvChi-R	tggtggtggtggtgctcgagTTATTGAATAGTGAAATTA
VF	CTCGAGCACCACCACCACCACCACTGA
VR	CATATGTATATCTCCTTCTTAAAGTTAAACAAAATTATTTCTAGAGGG

**Table 2:** The inhibition zone diameters of hydrolyzed products toward four pathogenic bacteria by disk filter paper assay.

Sample	Common Pathogens			Aquatic Product Pathogen
	Gram-Negative	Gram-Positive	Gram-Negative	Gram-Negative
	*E. coli*	*S. aureus*	*S. typhi* 50071	*A. hydrophila*
0 min	1.23 ± 0.10mm	N.D.	1.40 ± 0.02 mm	N.D.
20 min	2.26 ± 0.04 mm	3.14 ± 0.06 mm	3.49 ± 0.13 mm	3.63 ± 0.05 mm
40 min	2.31 ± 0.12 mm	2.56 ± 0.15 mm	1.17 ± 0.06 mm	1.30 ± 0.11 mm
60 min	N.D.	N.D.	1.12 ± 0.12 mm	1.13 ± 0.07 mm

N.D. indicates no detectable inhibition zone.

## Data Availability

The original contributions presented in this study are included in the article/Supplementary Materials. Further inquiries can be directed to the corresponding author.

## Supplementary Materials

The following supporting information can be downloaded at: https://www.mdpi.com/article/10.3390/foods15030575/s1, Table S1: 16S rDNA sequence-based identification of chitosan-degrading strains isolated from shrimp samples; Table S2: Comparison of enzymatic properties of chitosanases from different sources.; Figure S1: The chitosanase activities of isolated strains; Figure S2: The DNA sequences and the amino acids sequence of ByChi used in this study (the sequence in red represents the signal peptide); Figure S3: The predicted signal peptide by SignalP analysis. References [14,16,24,26,27,28,29] are cited in the supplementary materials.

## References

[1] Latgé J.P. (2007). The cell wall: A carbohydrate armour for the fungal cell. Mol. Microbiol..

[2] Aam B.B., Heggset E.B., Norberg A.L., Sørlie M., Vårum K.M., Eijsink V.G. (2010). Production of chitooligosaccharides and their potential applications in medicine. Mar. Drugs.

[3] Sugimoto M., Morimoto M., Sashiwa H., Saimoto H., Shigemasa Y. (1998). Preparation and characterization of water-soluble chitin and chitosan derivatives. Carbohyd. Polym..

[4] El-Araby A., Janati W., Ullah R., Ercisli S., Errachidi F. (2024). Chitosan, chitosan derivatives, and chitosan-based nanocomposites: Eco-friendly materials for advanced applications (a review). Front. Chem..

[5] Tsigos I., Martinou A., Kafetzopoulos D., Bouriotis V. (2000). Chitin deacetylases: New, versatile tools in biotechnology. Trends Biotechnol..

[6] Zhang W., Zhou J., Gu Q., Sun R., Yang W., Lu Y., Wang C., Yu X. (2022). Heterologous expression of GH5 chitosanase in *Pichia pastoris* and antioxidant biological activity of its chitooligosacchride hydrolysate. J. Biotechnol..

[7] Kim S.K., Rajapakse N. (2005). Enzymatic production and biological activities of chitosan oligosaccharides (COS): A review. Carbohyd. Polym..

[8] Sudharshan N.R., Hoover D.G., Knorr D. (1992). Antibacterial action of chitosan. Food Biotechnol..

[9] Kim J.Y., Lee J.K., Lee T.S., Park W.H. (2003). Synthesis of chitooligosaccharide derivative with quaternary ammonium group and its antimicrobial activity against *Streptococcus mutans*. Int. J. Biol. Macromol..

[10] Jeon Y.J., Kim S.K. (2000). Production of chitooligosaccharide using an ultrafiltration membrane reactor and their antibacterial activity. Carbohyd. Polym..

[11] No H.K., Park N.Y., Lee S.H., Hwang H.J., Meyers S.P. (2002). Antibacterial activities of chitosans and chitosan oligomers with different molecular weights on spoilage bacteria isolated from tofu. J. Food Sci..

[12] Cantarel B.L., Coutinho P.M., Rancurel C., Bernard T., Lombard V., Henrissat B. (2009). The Carbohydrate-Active EnZymes database (CAZy): An expert resource for glycogenomics. Nucleic Acids Res..

[13] Su H., Sun J., Jia Z., Zhao H., Mao X. (2022). Insights into promiscuous chitosanases: The known and the unknown. Appl. Microbiol. Biot..

[14] Wang Y., Mo H., Hu Z., Liu B., Zhang Z., Fang Y., Hou X., Liu S., Yang G. (2023). Production, characterization and application of a novel chitosanase from marine bacterium *Bacillus paramycoides* BP-N07. Foods.

[15] Wang J., Wang P., Zhu M., Chen W., Yu S., Zhong B. (2022). Overexpression and biochemical properties of a GH46 chitosanase from marine *Streptomyces hygroscopicus* R1 suitable for chitosan oligosaccharides preparation. Front. Microbiol..

[16] Xu Y., Wang H., Zhu B., Yao Z. (2024). Biochemical characterization and elucidation action mode of a new endolytic chitosanase for efficient preparation of chitosan oligosaccharides. Biomass Convers. Bior..

[17] Bhoopal B., Dokku S., Sk A., Gopi P., Samudrala R., Musti J.S., Appa R.P. (2021). New class of chitosanase from *Bacillus amyloliquefaciens* for the generation of chitooligosaccharides. J. Agric. Food Chem..

[18] Yang G., Sun H., Cao R., Liu Q., Mao X. (2020). Characterization of a novel glycoside hydrolase family 46 chitosanase, Csn-BAC, from *Bacillus* sp. MD-5. Int. J. Biol. Macromol..

[19] Singh R., Sharmila D. (2015). Antimicrobial effects of aqueous butanolic extract of *Saraca indica* (Linn). Res. J. Pharm. Biol. Chem. Sci..

[20] El-Sayed S.T., Ali A.M., El-Sayed E.M., Shousha W.G., Omar N.I. (2017). Characterization and potential antimicrobial effect of novel chitooligosaccharides against pathogenic microorganisms. J. Appl. Pharm. Sci..

[21] Chaudhry G.S., CS T., Zin N.A.M., Sung Y.Y., Muhammad T.S.T., AWM E. (2022). Antibacterial activity of Chito-oligosaccharides derived from Fish Scales. Res. J. Pharm. Tech..

[22] Takasuka T.E., Bianchetti C.M., Tobimatsu Y., Bergeman L.F., Ralph J., Fox B.G. (2014). Structure-guided analysis of catalytic specificity of the abundantly secreted chitosanase SACTE_ 5457 from *Streptomyces* sp. SirexAA-E. Proteins.

[23] Lyu Q., Shi Y., Wang S., Yang Y., Han B., Liu W., Jones D.N.M., Liu W. (2015). Structural and biochemical insights into the degradation mechanism of chitosan by chitosanase OU01. BBA-Gen Subj..

[24] Aktuganov G.E., Safina V.R., Galimzianova N.F., Gilvanova E.A., Kuzmina L.Y., Melentiev A.I., Baymiev A.H., Lopatin S.A. (2022). Constitutive chitosanase from *Bacillus thuringiensis* B-387 and its potential for preparation of antimicrobial chitooligomers. World J. Microb. Biot..

[25] Grossman T.H., Kawasaki E.S., Punreddy S.R., Osburne M.S. (1998). Spontaneous cAMP-dependent derepression of gene expression in stationary phase plays a role in recombinant expression instability. Gene.

[26] Wang Y., Li D., Liu M., Xia C., Fan Q., Li X., Lan Z., Shi G., Dong W., Li Z. (2021). Preparation of active chitooligosaccharides with a novel chitosanase Aq CoA and their application in fungal disease protection. J. Agric. Food Chem..

[27] Khayrova A., Lopatin S., Shagdarova B., Sinitsyna O., Sinitsyn A., Varlamov V. (2022). Evaluation of antibacterial and antifungal properties of low molecular weight chitosan extracted from *Hermetia illucens* relative to crab chitosan. Molecules.

[28] Zhao X.P., Liu J., Sui Z.J., Xu M.J., Zhu Z.Y. (2022). Preparation and antibacterial effect of chitooligosaccharides monomers with different polymerization degrees from crab shell chitosan by enzymatic hydrolysis. Biotechnol. Appl. Bioc..

[29] Mengíbar M., Ganan M., Miralles B., Carrascosa A.V., Martínez-Rodriguez A.J., Peter M.G., Heras A. (2022). Antibacterial activity of products of depolymerization of chitosans with lysozyme and chitosanase against *Campylobacter jejuni*. Carbohyd. Polym..

[30] Hao W., Li K., Li P. (2021). Review: Advances in preparation of chitooligosaccharides with heterogeneous sequences and their bioactivity. Carbohyd. Polym..

[31] Tsai G.J., Wu Z.Y., Su W.H. (2000). Antibacterial activity of a chitooligosaccharide mixture prepared by cellulase digestion of shrimp chitosan and its application to milk preservation. J. Food Prot..

[32] Yu D., Feng J., You H., Zhou S., Bai Y., He J., Cao H., Che Q., Guo J., Su Z. (2022). The microstructure, antibacterial and antitumor activities of chitosan oligosaccharides and derivatives. Mar. Drugs.

[33] Li K., Xing R., Liu S., Qin Y., Yu H., Li P. (2014). Size and pH effects of chitooligomers on antibacterial activity against *Staphylococcus aureus*. Int. J. Biol. Macromol..

[34] Peter E., João C.F., Eulália P., Manuela E.P., Malcata F.X. (2008). Atomic force microscopy study of the antibacterial effects of chitosans on *Escherichia coli* and *Staphylococcus aureus*. Ultramicroscopy.

[35] Helander I.M., Nurmiaho-Lassila E.L., Ahvenainen R., Rhoades J., Roller S. (2001). Chitosan disrupts the barrier properties of the outer membrane of gram-negative bacteria. Int. J. Food Microbiol..

[36] Kern N.R., Lee H.S., Wu E.L., Park S., Vanommeslaeghe K., MacKerell A.D., Klauda J.B., Jo S., Im W. (2014). Lipid-linked oligosaccharides in membranes sample conformations that facilitate binding to oligosaccharyltransferase. Biophys. J..

[37] Hussain A., Ong E.B.B., Balaram P., Ismail A., Kien P.K. (2023). Deletion of *Salmonella enterica* serovar Typhi tolC reduces bacterial adhesion and invasion toward host cells. Front. Microbiol..

