# Supplementary material for: Enzymatic Properties of Chitosanase from Bacillus velezensis YB1534 and Antibacterial Activity of Its Oligosaccharide Products
Source: Foods. 2026 Feb 5;15(3):575. doi: 10.3390/foods15030575 (PMC12897138; doi:10.3390/foods15030575)
Supplement: Supplementary file 1 [file foods-15-00575-s001.zip › foods-4124445-supplementary.pdf]

Supplementary Material

Enzymatic Properties of *Bacillus velezensis* YB1534 Chitosanase and Antibacterial Activity of Its Oligosaccharide Products

Yiwei Dai[1,*], Huiru Zhao[1], Yingxi Chen[1], Xinping Lin[1], Sufang Zhang[1], Chaofan Ji[1]

1 State Key Laboratory of Marine Food Processing & Safety Control, National Engineering Research Center of Seafood, School of Food Science and Technology, Dalian Polytechnic University, Dalian, Liaoning, 116034, China

* Corresponding author:

Yiwei Dai, Tel.:+86-182-6228-4612; E-mail address: ywdai6228@126.com

Table S1. 16S rDNA sequence-based identification of chitosan-degrading strains isolated from shrimp samples.

Strains	Scientific name	Indentity
XX-1	*Acinetobacter calcoaceticus* strain A46	100.00%
XX-2	*Acinetobacter baumannii* strain B	99.86%
XX-3	*Acinetobacter sp.strain* TH-S-11-1	99.86%
XX-4	*Bacterium strain* QLS201807OPB3	99.79%
XX-5	*Bacterium strain* XFZ4	99.65%
XX-6	*Bacterium strain* BS0171	99.72%
XX-7	*Bacterium strain* BS1876	99.51%
XX-8	*Bacterium strain* BS0508	100.00%
XX-9	*Cedecea davisae* DSM 4568	99.63%
XX-10	*Bacterium strain* WG90917	99.79%
YB1534	*Bacillus velezensis* strain K-2	99.93%
YB1536	*Bacillus amyloliquefaciens* strain B10	100.00%
YB1531	*Bacillus velezensis* strain B49	100.00%
YB2433	*Bacillus amyloliquefaciens* subsp.*plantarum* strain HK-2-26	100.00%
YB1514	*Bacillus cereus* strain BA6-1	100.00%
MJD11B	*Aspergillus minisclerotigenes*	99.19%
MJD28B	*Aspergillus oryzae* isolate TURS11	99.67%

Table S2. Comparison of enzymatic properties of chitosanases from different sources.

Chitosanase source	Molecular weight	Optimal temperature	Optimal pH	Enzyme activity	Minimum DP of products	Antimicrobial activity of products (MIC)	Reference
B. paramycoides BP-N07	37 kDa	50℃	6.0	8133.17±47.83 U/mg	2	-	[14]
Kitasatospora setae KM-6054	31.07 kDa	60℃	5.0	241.39 U/mg	2	-	[16]
Aquabacterium sp. A7-Y	50.7 kDa	40℃	5.0	18 U/mg	3	*Magnaporthe oryzae*<br>*Fusarium oxysporum*	[26]
B. thuringiensis var. *dendrolimus* B-387	40 kDa	55℃	6.5	-	2	*E. coli* (70 ± 8 μg/mL)<br>*Pseudomonas aeruginosa* (> 2000 μg/mL)<br>*Enterobacter cloaceae* (60 ± 3 μg/mL)<br>*B. cereus* (1610 ± 150 μg/mL)	[24]
Trichoderma harzianum<br>*Myceliophthora thermophila*	42 kDa<br>43 kDa	-	-	2000 U/g<br>1500 U/g	Average M_w 6-21 kDA	*S. epidermidis* (62.5 μg/mL)<br>*E. coli* (＞500 μg/mL)	[27]
Commercial chitosanase	-	50 ℃	5.2	102.53 U/g	2	*Salmonella* (64 mg/mL)	[28]

Commercial chitosanase from *Streptomyces griseus*	-	-	5.7	-	-	*Campylobacter jejuni*	[29]
B. velezensis YB1534	31 kDa	50℃	6.0	338.2 U/mg	2	*E. coli* (0.625 mg/mL)<br>*S. aureus* (0.313 mg/mL)<br>*S. typhi* 50071 (1.25 mg/mL)<br>*A. hydrophila* (0.625 mg/mL)	This study

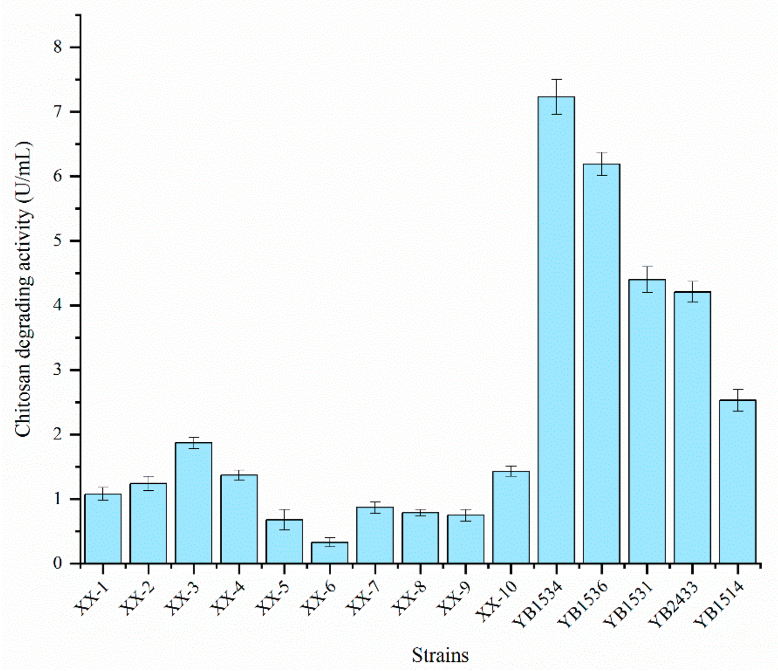

Figure S1. The chitosanase activities of isolated strains.

```
ORIGIN
1        ATGAAAATCA GCTTGAAGAA AAAAGCAGGT TTTTGGAAGA AGACGGCGGT TTCGTCACTT
61       ATTTTCACCA TGTTTTTTAC CCTGATGATG AGCGGTACGG TTTTTGCGGC CGGGCTGAAT
121      ACGGATCAGA AGCGCCGGGC GGAACAGCTG ACCAGCATCT TTGAAAACGG AAAGACGGAA
181      ATCCAATACG GATATGTTGA AGCGTTGGAT GACGGAAGAG GTTACACTTG CGGGCGGGCC
241      GGCTTTACGA CGGCTACCGG AGATGCGCTG GAAGTAGTCG AAGTATACAC GAAAGCGGTG
301      CCGAATAACA AATTGAAAAA GTATTTGCCT GAATTGCGGC GTCTTGCGAA GGACGAAAGC
361      GATGACATCA GCAATCTGAA AGGATTCGCT TCTGCCTGGC GCTCACTTGG CAATGATAAA
421      GCCTTCCGCG CTGCCCAAGA TAAGGTAAAT GACAGCTTGT ATTATCAGCC GGCGATGAAA
481      CGTTCAGAAA ATGCCGGACT GAAAACGGCC TTGGCAAAAG CAGTGATGTA CGATACGGTG
541      ATTCAGCATG GCGACGGCGA TGATCCAGAC TCCTTTTATG CCCTGATTAA ACGCACGAAC
601      AAAAAAATGG GCGGGTCACC GAAAGACGGA ACTGACGAGA AGAAATGGCT CAATAAATTC
661      TTGGATGTGC GCTATGACGA TCTGATGAAT CCGTCAGATG AGGACACTCA GGATGAATGG
721      AGAGAATCGG TTGCCCGTGT CGACGTTTTC CGCGATATTG TCAAAGAGAA GAACTACAAT
781      TTAAACGGGC CGATTCATGT CCGGTCAAGC GAATACGGTA ATTTCACTAT TCAATAA
```

```
ORIGIN
1        MKISLKKKAG FWKKTAVSSL IFTMFFTLMM SGTVFAAGLN TDQKRRAEQL TSIFENGKTE
61       IQYGYVEALD DGRGYTCGRA GFTTATGDAL EVVEVYTKAV PNNKLKKYLP ELRRLAKDES
121      DDISNLKGFA SAWRSLGNDK AFRAAQDKVN DSLYYQPAMK RSENAGLKTA LAKAVMYDTV
181      IQHGDGDDPD SFYALIKRTN KKMGGSPKDG TDEKKWLNKF LDVRYDDLMN PSDEDTQDEW
241      RESVARVDVF RDIVKEKNYN LNGPIHVRSS EYGNFTIQ
```

Figure S2 The DNA sequences and the amino acids sequence of ByChi used in this study (the sequence in red represents the signal peptide).

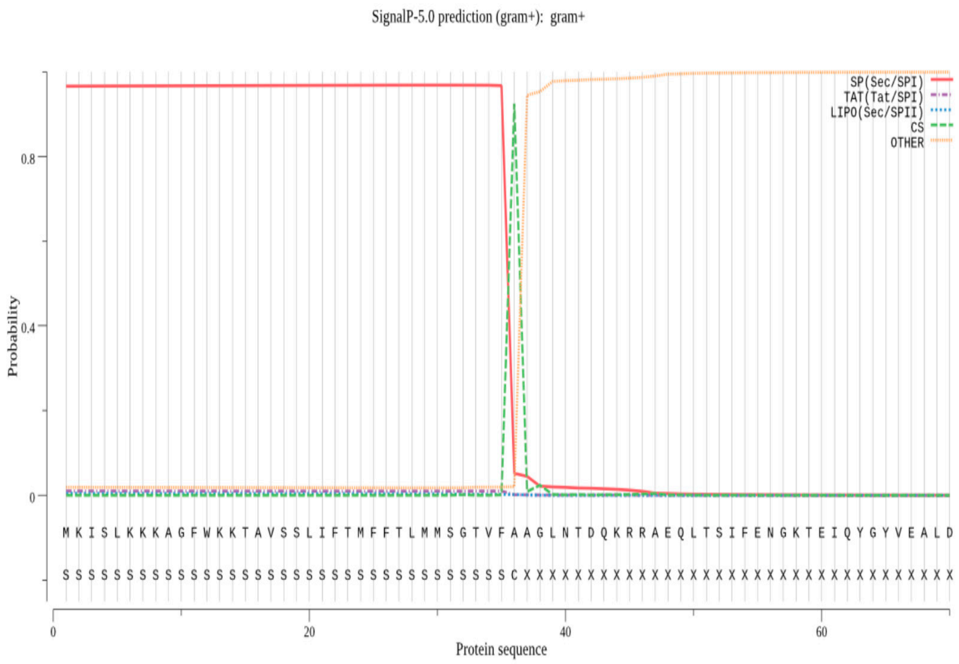

Figure S3 The predicted signal peptide by SignalP analysis.

Reference

[14] Wang, Y., Mo, H., Hu, Z., Liu, B., Zhang, Z., Fang, Y., Hou, X., Liu, S., & Yang, G. Production, characterization and application of a novel chitosanase from marine bacterium *Bacillus paramycoides* BP-N07. Foods 2023, 12, 3350.

[16] Xu, Y., Wang, H., Zhu, B., & Yao, Z. Biochemical characterization and elucidation action mode of a new endolytic chitosanase for efficient preparation of chitosan oligosaccharides. Biomass Convers Bior 2024, 14, 18897-18905.

[24] Aktuganov, G. E., Safina, V. R., Galimzianova, N. F., Gilvanova, E. A., Kuzmina, L. Y., Melentiev, A. I., Baymiev, A. H., & Lopatin, S. A. Constitutive chitosanase from *Bacillus thuringiensis* B-387 and its potential for preparation of antimicrobial chitooligomers. World J Microb Biot 2022, 38, 167.

[26] Wang, Y., Li, D., Liu, M., Xia, C., Fan, Q., Li, X., Lan, Z., Shi, G., Dong, W., Li, Z., & Cui, Z. Preparation of active chitooligo-saccharides with a novel chitosanase Aq CoA and their application in fungal disease protection. J Agric Food Chem 2021, 69, 3351-3361.

[27] Khayrova, A., Lopatin, S., Shagdarova, B., Sinitsyna, O., Sinitsyn, A., & Varlamov, V. Evaluation of antibacterial and antifungal properties of low molecular weight chitosan extracted from *Hermetia illucens* relative to crab chitosan. Molecules 2022, 27(2).

[28] Zhao, X. P., Liu, J., Sui, Z. J., Xu, M. J., & Zhu, Z. Y. Preparation and antibacterial effect of chitooligosaccharides monomers with different polymerization degrees from crab shell chitosan by enzymatic hydrolysis. Biotechnol Appl Bioc 2022, 70(1), 164-174.

[29] Mengíbar, M., Ganan, M., Miralles, B., Carrascosa, A. V., Martínez-Rodriguez, A. J., Peter, M. G., & Heras, A. Antibacterial activity of products of depolymerization of chitosans with lysozyme and chitosanase against *Campylobacter jejuni*. Carbohyd Polym 2022, 84(2), 844-848.
